# Assessment of MicroRNAs Associated with Tumor Purity by Random Forest Regression

**DOI:** 10.3390/biology11050787

**Published:** 2022-05-21

**Authors:** Dong-Yeon Nam, Je-Keun Rhee

**Affiliations:** School of Systems Biomedical Science, Soongsil University, Seoul 06987, Korea; namdongyeon@soongsil.ac.kr

**Keywords:** tumor purity, tumor microenvironment, microRNA, machine learning, random forest regression

## Abstract

**Simple Summary:**

Cancer is a disease with high mortality and recurrence rates. To understand cancer biology, it is important to accurately determine the proportion of tumor and non-tumor cells in tumor tissues. In this study, the proportion of tumor cells in tumor tissues was predicted using miRNA expression data that had not been sufficiently studied before. Using a random forest regression model, the tumor purity was predicted accurately, and subsequent investigations into the association between the informative microRNAs and tumor purity could be conducted.

**Abstract:**

Tumor purity refers to the proportion of tumor cells in tumor tissue samples. This value plays an important role in understanding the mechanisms of the tumor microenvironment. Although various attempts have been made to predict tumor purity, attempts to predict tumor purity using miRNAs are still lacking. We predicted tumor purity using miRNA expression data for 16 TCGA tumor types using random forest regression. In addition, we identified miRNAs with high feature-importance scores and examined the extent of the change in predictive performance using informative miRNAs. The predictive performance obtained using only 10 miRNAs with high feature importance was close to the result obtained using all miRNAs. Furthermore, we also found genes targeted by miRNAs and confirmed that these genes were mainly related to immune and cancer pathways. Therefore, we found that the miRNA expression data could predict tumor purity well, and the results suggested the possibility that 10 miRNAs with high feature importance could be used as potential markers to predict tumor purity and to help improve our understanding of the tumor microenvironment.

## 1. Introduction

MicroRNAs (miRNAs) are ~22-nt RNAs that regulate gene expression at the post-transcriptional level [1] and are closely related to a variety of complex diseases [2]. Understanding the roles of miRNAs in complex diseases can help us to understand the molecular mechanisms of the diseases and to detect biomarkers for diseases and their treatment responses. However, traditional wet-lab experiments to determine the relationships between miRNAs and complex diseases are costly and time-consuming. Thus, in recent years, many computational prediction models have been developed based on high-throughput datasets [3].

MiRNAs are also involved in modifying multiple aspects of tumor development [4]. For example, hepatocellular carcinoma can be developed by the disruption of a feedback loop between miR-122 and c-Myc [5]. Moreover, the role of miR-17-92 clusters is well documented in several tumors [6,7]. In addition, miRNAs participate in remodeling the components of tumor microenvironments [8,9]. For instance, cancer-associated fibroblasts (CAFs) commonly found in tumor microenvironments are major components that support tumor development, and the conversion of normal fibroblasts to CAFs can be related to an alteration in miRNA expression [10]. In addition, miR-31 regulates the overexpression of the *SATB2* gene in CAFs, which increases tumor cell migration and invasion [11]. In addition, the downregulation of miR-101, targeting *CXCL12*, which plays an important role in CAF, can inhibit proliferation and metastasis in lung cancer [12].

Generally, tumor cells coexist with non-cancerous cells such as immune cells, fibroblasts, and blood vessels in the tumor microenvironment [13]. Recently, it has been shown that both cancerous and non-cancerous cells play important roles in cancer biology [14]. Thus, with the increasing importance of the tumor microenvironment, it has become important to determine the ratio of cancerous to non-cancerous cells and the tumor purity in tumor samples.

Tumor purity has previously been determined by pathologists, but with the development of high-throughput technologies and the production of large numbers of datasets, tumor purity has come to be estimated by computational methods. The most representative methods are ABSOLUTE [15] and ESTIMATE [16]. ABSOLUTE aims to extract the absolute copy number of cancer cells from admixed populations of cells and estimates tumor purity and ploidy with precomputed statistical models for recurrently observed genomes in cancer cells. On the other hand, ESTIMATE utilizes gene expression data. This method defines stromal-related and immune-related signature genes and infers the tumor purity by combining the stromal and immune scores. Moreover, some recent studies have determined tumor purity using machine learning methods. Using gene-expression data, Koo and Rhee showed that the tumor purity could be predicted well by various machine learning-based regression models [17], and Li et al. developed a tumor purity prediction model based on XGBoost, a supervised machine learning method [18]. RF_purity uses DNA methylation data based on random forest regression.

However, a tumor purity prediction method using miRNAs has not yet been developed. Because miRNAs can affect the tumor microenvironment by regulating gene expression [9], it may be possible to estimate tumor purity using miRNA expression. Here, we built a tumor purity prediction model based on a supervised machine learning method using The Cancer Genome Atlas (TCGA) datasets for 17 tumor types with more than 300 samples per type. This study aimed to measure the predictive performance of miRNA expression and to verify the miRNAs associated with tumor purity using feature-importance scores. Moreover, by investigating the biological roles of genes related to these miRNAs, we investigated the potential roles of these miRNAs in the tumor and tumor microenvironment.

## 2. Materials and Methods

### 2.1. Dataset Description for Model Construction

MicroRNA expression quantification (stem loop) was downloaded from the TCGA Genomic Data Commons (GDC) repository UCSC Xena (https://xenabrowser.net/) [19]. From the database, tumor types with more than 300 samples per tumor type were selected, and a total of 9062 samples across 17 human tumor types were used for the experiments (Table 1). These datasets were represented as 1881 miRNA expression values (log2(RPM+1)) per sample.

Tumor purity values for the samples were obtained from the consensus measurement of purity estimation (CPE) scores calculated by Aran et al. [20]. However, the stomach adenocarcinoma (STAD) tumor type was excluded because there were no CPE values in the data reported by Aran et al. Finally, after excluding samples with missing information, 16 tumor types with 7250 samples were used in our experiments.

### 2.2. Random Forest Regression and Optimizing Parameters

The random forest regression (RFR) algorithm was used to predict tumor purity from the miRNA expression values. Random forest is an ensemble method of learning multiple decision trees, a combination of tree predictors where each tree independently relies on the values of sampled random vectors and has the same distribution for all trees in the forest [21]. Random forest has the advantages of significantly higher accuracy, fast learning and testing algorithms, and good generalization performance through randomization.

To learn and evaluate the models, the 7250-sample dataset was split into a training dataset (70%) and a test dataset (30%). The parameters of the model were tuned by a grid search with 10-fold cross-validation (GridSearchCV) using the training dataset. The parameter values with the best performance were selected by varying the parameter values. The final maximum depth of the trees was 20, and the number of trees was 500. The prediction performance was evaluated using the mean square error (MSE) of the target values (CPE), as follows:

(1) $$\mathrm{MSE}=\frac{1}{N}\overset{N}{\underset{i=1}{\sum }}{\left(f_{i}-y_{i}\right)}^{2}$$

where *N* is the number of samples, *f_i_* is the predicted tumor purity for sample *i*, and *y_i_* is the target value for sample *i*. All the machine learning experiments were implemented using the Python scikit-learn library [22].

### 2.3. Feature Importance of miRNAs with Random Forest Regression

The feature importance as a node-impurity reduction weighted by the probability that each node reaches the node representing the miRNA can be calculated using the random forest regression model. The node probability was obtained by dividing the number of samples that reached the node by the total number of samples. To verify the relationship between tumor purity and miRNA with high feature importance, the Pearson correlation coefficient (PCC) between the tumor purity and expression levels of the miRNAs with high feature-importance scores was measured.

Eight regression models, including support vector regression (SVR), K neighbors regression (KNR), multilayer perceptron (MLP), elastic net regression (ElasticNet), linear regression (linear), least absolute shrinkage and selection operator (Lasso), and ridge regression (Ridge), were used to determine whether the predicted performance with few miRNAs was similar to those obtained using all miRNAs and to determine the extent of changes in the model performance as the number of miRNAs with high feature importance increased. For these models, excluding random forest regression, the optimal parameters were obtained using a grid search with 10-fold cross-validation (GridSearchCV) and applied to the models. The parameters selected for these models are listed in Table S1.

### 2.4. Target Gene Prediction for miRNA with High Feature Importance

For the target prediction of the miRNAs with high feature-importance scores, TargetScan [23] and miRDB [24] were used. For miRDB, the target prediction score was 50 to 100 for all predictors, but only genes with a prediction score of 80 or higher were selected as the real target according to the authors’ guidelines. The selected target genes were obtained by the intersection of the prediction results of the tools TargetScan and miRDB.

### 2.5. Enrichment Analysis of Target Genes

Enrichr (https://maayanlab.cloud/Enrichr/) (accessed on 3 November 2021) [25] was used to assess which Kyoto Encyclopedia of Genes and Genomes (KEGG) pathways [26] and biological processes of gene ontology (GO) were enriched by the target genes. The enrichment terms with a low-adjusted *p*-value < 0.01 were selected as indicators using the Benjamini–Hochberg method for correction for multiple hypothesis testing.

### 2.6. Validation with Independent Datasets

TCGA tumor types, which were not used for model development (the number of samples was lower than 300), were used for the validation experiments (Table 2). The datasets were also downloaded from the TCGA Genomic Data Commons (GDC) repository UCSC Xena [19]. The number of samples was 1958 across 16 human tumor types. The data were preprocessed using the same process described in Section 2.1 and fed into the random forest model trained using the top 10 miRNAs chosen by the feature-importance scores as a test set.

Moreover, to further validate our results, the miRNA expression and purity data in Pan-Cancer Analysis of Whole Genomes (PCAWG) (specimen centric) were obtained from UCSC Xena [19]. The PCAWG miRNA expression (UQ normalized) consisted of 1524 samples and 1864 miRNA expression values. By integrating the miRNA expression data and the purity data based on the samples, a total of 771 samples were used in the validation experiment.

PCAWG miRNA data were composed of mature miRNA expression, whereas the TCGA miRNA expression involved precursor forms. Thus, the RFR model was re-trained using the PCAWG mature miRNA expression data with the top 10 miRNAs using the feature-importance scores by splitting them into a training set (70%) and a test set (30%); we then evaluated the prediction performance.

## 3. Results

### 3.1. Predictive Performance of Random Forest Regression Model

**Figure 1:**
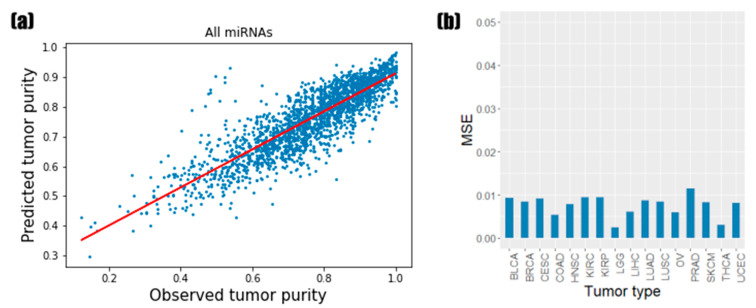
Prediction results of the tumor purity using miRNA expression. (**a**) Scatterplots of tumor purity values across the 16 tumor types of the test samples. The red line is the regression line. (**b**) Bar plots of the test mean square error (MSE) value for each tumor type.

Prior to predicting the tumor purity by random forest regression, a grid search with 10-fold cross-validation was performed to determine the optimal parameters. As the result of the grid search with 10-fold cross-validation, a maximum depth of 20 trees and a number of 500 trees were finally selected. We trained the random forest regressor using the selected parameters and predicted the test performance using the remaining test sets. Figure 1a presents the experimental results of the test data for the 16 tumor types, which shows that there is a high positive correlation between the observed tumor purity values (CPE) and the values predicted by random forest regression (PCC = 0.8626). The MSE of the random forest regression for the test data was 0.0067. When comparing the prediction results for each tumor type, brain lower-grade glioma (LGG) showed the best results at 0.0024, and prostate adenocarcinoma (PRAD) was the worst at 0.0114 (Figure 1b). The random forest regressor showed good prediction results regardless of the tumor type, although the predictive performances were slightly different for each tumor type.

### 3.2. Identification of the Informative miRNAs with Feature Importance

Feature-importance scores were acquired from random forest regression. We examined the feature-importance scores to identify miRNAs that are important in tumor purity estimation. Table 3 shows the top 10 miRNAs ranked by their feature-importance scores. The miRNA with the highest feature-importance score was hsa-mir-155, which is required for CD8(+) T-cell responses to cancer [27]. Similarly, it has been shown that hsa-mir-155 overexpression enhances the anti-tumor response [27]. The second most abundant miRNA, hsa-mir-4772, has been reported as a prognostic biomarker for tumor recurrence in patients with stage II and stage III colon cancer [28]. Third, hsa-mir-142 was downregulated in hepatocellular carcinoma (HCC), and its expression level decreased as the disease worsened [29]. The overexpression of hsa-mir-142 significantly suppresses HCC cell migration and invasion [29]. Moreover, hsa-mir-150 also acts as a biomarker for hepatitis B virus-related HCC [30]. The serum hsa-mir-150 expression is considerably reduced in HCC patients compared to healthy controls [30]. In addition, hsa-miR-150 is noticeably downregulated in advanced cutaneous T-cell lymphoma, and the downregulation of hsa-miR-150 is related to tumor invasion and metastasis [31]. hsa-miR-223 inhibits the expression of various genes involved in inflammation and modulates tumor malignancy [32,33]. The expression of hsa-miR-200c and hsa-miR-200a modulates the epithelial-to-mesenchymal transition (EMT) of various tumor types [34,35,36]. In particular, hsa-miR-200c has recently been shown to play a role in interactions between tumor cells and macrophages [37]. Moreover, a recent study showed that has-miR-200a activates the expression of PD-L1 and dysregulates anti-tumor immunity [38]. has-miR-141 is a member of the miR-200 family, and this miRNA is located next to hsa-miR-200c on chromosome 12. Thus, the hsa-miR-141 also has a similar role to other miRNAs within the miR-200 family. The abnormal expression of miR-92a family members is found in various malignant tumors such as oral squamous cell carcinoma and acute myeloid leukemia [39,40]. miR-22 also plays a biological role in tumor angiogenesis, EMT, proliferation, migration, invasion, etc. [41].

Furthermore, we calculated the PCC between miRNA expression and tumor purity (CPE). It was noted that the PCCs for the top five miRNAs were negative.

### 3.3. Prediction Results of Models Based on miRNA Feature Importance

**Figure 2:**
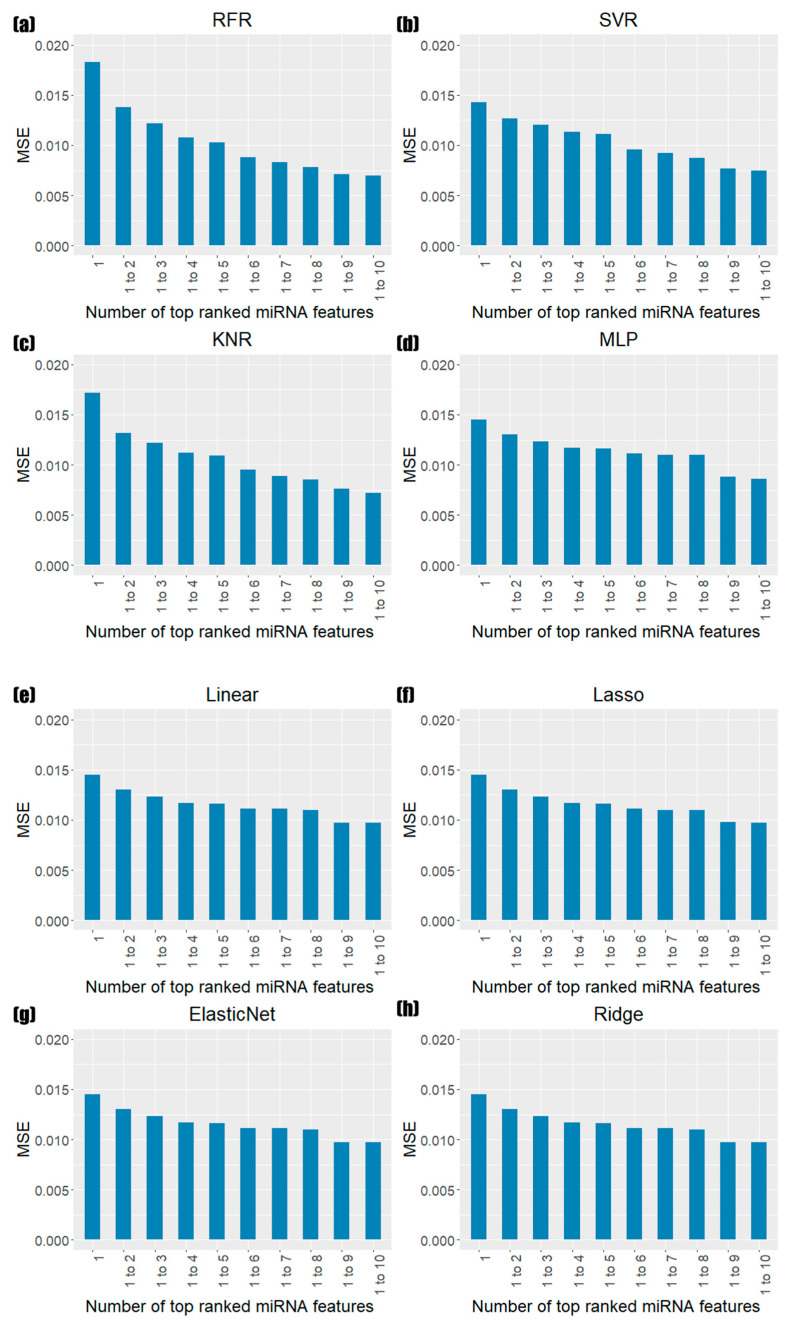
Bar plot test of the MSE by increasing miRNA features from the top one to the tenth using eight regression models. These are (**a**) random forest regression (RFR), (**b**) support-vector regression (SVR), (**c**) K neighbors regression (KNR), (**d**) multilayer perceptron (MLP), € linear regression (Linear), (**f**) least absolute shrinkage and selection operator (Lasso), (**g**) elastic net regression (ElasticNet), and (**h**) ridge regression (Ridge).

To assess the significance of miRNAs with high feature-importance scores, we constructed eight machine learning models to predict the tumor purity using only miRNAs. The prediction performance of the eight models was estimated by increasing the miRNA features one by one according to the feature-importance ranking (Figure 2).

The test MSEs using only hsa-mir-155, which is the top miRNA in terms of features, were 0.0143 (SVR) to 0.0183 (RFR) across the 16 tumor types. Although the overall performance was not good in the very simple model, it improved as the number of highly ranked miRNA features increased. Using the top 10 miRNA features, the test MSEs ranged from 0.0070 (RFR) to 0.0099 (ElasticNet). Although the performance of RFR models using all miRNAs (0.0067) was better than that of the models using a small number of highly ranked miRNAs, the results showed that a small number of highly ranked miRNAs could significantly predict the tumor purity.

**Figure 3:**
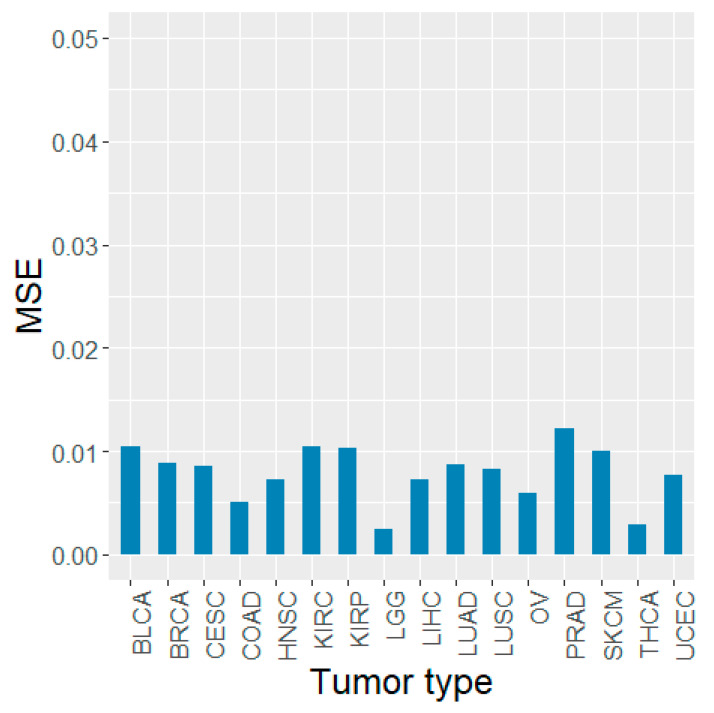
Test MSE values of the RFR model for the top 10 miRNAs in each tumor type.

In addition, for each tumor type, the test MSEs using only the 10 informative miRNAs were very similar to the prediction results obtained using all 1881 miRNAs (Figure 3). The tumor type that showed the best performance was the same as that obtained for all miRNAs, and the MSE was the same at 0.0024. The worst MSE was also observed in PRAD—the same tumor type as the result of all miRNAs. The MSE value was 0.0122. In addition, the other tumor types showed very similar MSE values, with differences ranging from 0.0000 to 0.0017. This indicates that miRNAs with high feature-importance scores had a great impact on tumor purity prediction, even with a small number of miRNAs.

### 3.4. Enrichment Analysis of Target Genes Predicted by Top Ranked miRNAs

For the further functional validation of the miRNAs ranked by feature importance, we obtained the target genes of miRNA predicted by miRDB version 6.0 and TargetScan version 8.0, and the target genes predicted by the two tools were intersected. A list of target genes is presented in Tables S2 and S3. We investigated which KEGG pathways and GO terms were enriched in the target genes. Table 4 shows the enrichment analysis using the target genes by miRNA (has-miR-155) based on the KEGG pathways with an adjusted *p*-value < 0.01; Table S4 represents the whole list of KEGG pathways with statistical significance (adjusted *p*-value < 0.05). The first ranked term was the interleukin (IL)-17 signaling pathway, which activates the mitogen-activated protein kinase (MAPK), CCAAT-enhancer-binding protein β (C/EBPβ), and nuclear factor κB (NF-κB) pathways. This activates the transcription of downstream target genes, including proinflammatory cytokines and chemokines [42]. Additionally, this IL-17 can act on tissue stem cells and promote the formation, growth, and metastasis of tumors [43]. Hepatitis B with the same *p*-value as the IL-17 signaling pathway was also confirmed in a previous study [44] to be closely related to hsa-mir-155, which could be demonstrated once again through the KEGG pathway. Additionally, the tumor necrosis factor (TNF) signaling pathway plays an important role in regulating immune responses and inducing inflammation [45]. Other immune-related pathways were identified, including the T-cell and B-cell receptor signaling pathways. Moreover, several obvious cancer pathways such as pathways in cancer, colorectal cancer, and pancreatic cancer were also highly ranked. The results suggest that has-mir-155, which ranked first in the feature-importance scores, is apparently associated with immunity and tumors, as we expected.

Likewise, we performed an enrichment analysis using the genes targeted by the top-three miRNAs (hsa-mir-155, hsa-mir-4772, and hsa-mir-142) (Table S5). Similarly, the target genes were mainly associated with immunity and cancer-related pathways, including pathways related to the extracellular matrix, such as the cytoskeleton and adherens junctions. This could be because miRNAs play an important role in cell–cell interactions in tumor microenvironments [46]. These results indicate that miRNAs can not only predict tumor purity but also have close relationships with tumor microenvironments.

We also conducted GO analyses of the target genes. Tables S6 and S7 show the enrichment results for the target genes of the top one and three miRNAs, respectively. As shown in Table S6, the two top-ranked terms were the regulation of transcription, DNA-templated (GO:0006355), and the regulation of transcription by RNA polymerase II (GO:0006357), which are all related to the regulation of transcription. In addition, some immune-related GO terms were verified, such as the cellular response to interleukin-6 (GO:0071354), which is a process that causes changes in the state or activity of the cell due to IL-6 stimulation, and the IL-6-mediated signaling pathway (GO:0070102), which is a series of molecular signals, starting with IL-6 binding to receptors on the cell surface. Interleukin-6 supports the specific differentiation of naïve CD4+ T cells [47] and causes the differentiation of CD8+ T cells into cytotoxic T cells [48]. Thus, IL-6 plays an important role in connecting innate and acquired immune responses [47]. Similarly, when examining genes targeted by the top three miRNAs, the regulation of transcription, the GO terms closely related to the nature of cancer in which cell growth and differentiation are abnormally regulated were found to have the lowest adjusted *p*-values. Moreover, several GO terms related to the extracellular matrix, including cytoskeleton and cell migration, were also detected (Table S7). The GO analysis again confirmed that the target genes were related to tumors, especially the tumor microenvironment.

### 3.5. Validation Using Other TCGA Tumor Types and PCAWG Data

**Figure 4:**
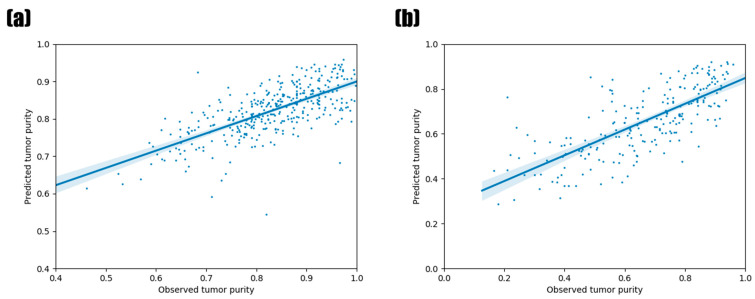
Prediction of tumor purity using validation datasets based on the top 10 miRNAs. (**a**) TCGA tumor types not used in our model construction. (**b**) PCAWG test dataset. The fitted line by linear regression is also shown with a 95% confidence interval. The plot was generated by regplot() in the seabon package of Python.

To verify the established RFR model and the significance of the top 10 miRNAs for tumor purity prediction, we employed the miRNA expression data of the other 16 tumor types (Table 2) not used for the model construction as a test set. Figure 4a shows the tumor purity prediction results. The PCC between the observed tumor purity value and the predicted value was 0.6982 (*p*-value = 1.60 × 10^−54^), and the test MSE was 0.0054. These were similar to the previous PCC and test MSE results on 16 tumor types, whose range of PCC in 16 tumor types was from 0.2876 to 0.8910 (median = 0.6937), while the MSE ranged from 0.0024 to 0.0122 (Figure 3). Thus, our RFR model could also predict the tumor purity accurately in the other 16 tumor types.

For the further validation of the relevance of the top 10 miRNAs, we utilized the miRNA expression data of PCAWG (Figure 4b). The RFR model was re-constructed with the training data from PCAWG, since they were the expression levels of the mature miRNAs in contrast to the TCGA data, which represented miRNA stem loops. Although the test MSE was 0.0164, a little higher than the results of the TCGA data, the PCC between the observed tumor purity value and the predicted tumor purity value was 0.7421 (*p*-value = 7.65 × 10^−42^), higher than the TCGA validation data.

## 4. Discussion

In this study, we constructed a random forest regression model, which is a supervised machine learning algorithm based on regression, to predict tumor purity. We trained it using miRNA expression profiles and confirmed that the model was trained well to search for a low MSE value, indicating the difference between the observed and predicted tumor purity values. The experimental results showed a positive correlation between the predicted and observed tumor purity values, indicating the high reliability of the predicted tumor purity value.

One of the advantages of random-forest-based models is that they provide feature-importance scores, reflecting the importance of each feature (miRNA) in predicting tumor purity as well as providing high predictive performance. Generally, many supervised machine learning algorithms are regarded as black-box models, meaning that they can perform well; however, it is difficult to explain why the results are produced. Our model identified the top-ranked miRNAs according to the feature-importance score, and we found that these were related to immune and cancer-related responses [27,28,29,30,31]. Thus, it was validated that miRNAs selected by the feature-importance scores actually had roles in tumor and tumor microenvironments, and, consequently, their expression could be associated with tumor purity.

In addition, we explored whether miRNAs with high feature-importance scores could predict tumor purity well. Our results validated that a small number of miRNAs can be used to make accurate tumor purity predictions, and the predictive performances did not show any apparent changes between the results for all miRNAs and the top 10 miRNAs. Moreover, we verified the target genes of the top-ranked miRNAs, which are also strongly associated with tumors and tumor microenvironments. Our results make the informative miRNAs worthy of further research as biomarker candidates.

Furthermore, through additional validation experiments, we not only showed that our model predicts tumor purity values well, even when using independent data, but also that the top-10 miRNAs were relevant for the tumor purity prediction. In particular, the PCAWA represents mature miRNA expression, not stem loops. Additionally, the target values (tumor purity) were estimated by copy-number alteration and SNVs, in contrast to the CPE values used in our RFR model training. Despite the incompatible aspects, the machine learning model accurately predicted the tumor purity using the miRNA expression levels.

One limitation of this study is that not all values of the parameters for the machine learning algorithms were tested to find the optimal parameters. In fact, testing all the possible cases of the continuous values would be too time-consuming; thus, we tried to find the rigorous parameter values by a grid search with cross-validation based on pre-selected hyperparameter values, but there exists the possibility for better parameters among the untested options.

In recent years, many computational models for miRNA–disease association prediction have been developed [49,50,51]. For example, a computational prediction model for miRNA–disease association, IMCMDA (Inductive Matrix Completion for MiRNA–Disease Association prediction), identified miRNAs related to colon tumors, kidney tumors, and lymphoma [49]. Another model based on the matrix completion, NCMCMDA (Neighborhood Constraint Matrix Completion for MiRNA–Disease Association prediction), could also uncover missing miRNA–disease associations [52]. Moreover, some studies predicted the potential associations between miRNAs and diseases using a deep belief network [53] and stacked autoencoder [54]. Although our model focused on the prediction of tumor purity and the identification of important miRNAs for tumor purity prediction, in future work, it will be necessary to develop a method of finding the direct relationships between the miRNAs and tumor development, progression, prognosis, etc. Furthermore, with the development of recent computational studies, the importance of non-coding RNAs (ncRNAs) has been uncovered. The ncRNAs interact with other molecules and make complex biological networks, consequently affecting biological functions. The integrative analysis with various non-coding RNAs can provide further insights into the potential roles of the non-coding RNAs in tumors. Moreover, further studies on building biological networks, including mRNA–miRNA–lncRNA interactions [55,56,57,58,59,60], and identifying the association between disease and the dysregulation of these networks will help us to identify potential biomarkers and improve our understanding of cancer biology.

## 5. Conclusions

In summary, we predicted tumor purity from miRNA expression data using random forest regression and identified informative miRNAs for the tumor purity prediction. Furthermore, we showed that the microRNAs had an influence on the tumor microenvironment and could be acted as biomarkers for predicting the tumor purity. To our knowledge, no study has predicted tumor purity using miRNA expression in the past. This study suggests the possibility that miRNAs act as important factors in the tumor microenvironment and could be a basis for research on the regulation of miRNAs in tumor microenvironments.

## Figures and Tables

**Table 1:** Abbreviation for tumor type and the number of genomic data commons (GDC) from The Cancer Genome Atlas (TCGA) microRNA (miRNA) expression datasets.

Tumor Type	Abbreviation	Number of Samples
Breast invasive carcinoma	BRCA	1202
Kidney renal clear cell carcinoma	KIRC	592
Uterine corpus endometrial carcinoma	UCEC	575
Thyroid carcinoma	THCA	573
Head and neck squamous cell carcinoma	HNSC	569
Lung adenocarcinoma	LUAD	564
Prostate adenocarcinoma	PRAD	551
Brain lower-grade glioma	LGG	530
Lung squamous cell carcinoma	LUSC	523
Ovarian serous cystadenocarcinoma	OV	498
Stomach adenocarcinoma *	STAD	477
Colon adenocarcinoma	COAD	461
Skin cutaneous melanoma	SKCM	452
Bladder urothelial carcinoma	BLCA	432
Liver hepatocellular carcinoma	LIHC	425
Kidney renal papillary cell carcinoma	KIRP	326
Cervical squamous cell carcinoma and Endocervical adenocarcinoma	CESC	312

* STAD was not used in these experiments because tumor purity information is not available for this tumor type.

**Table 2:** Abbreviation for tumor type and the number of genomic data commons (GDC) in The Cancer Genome Atlas (TCGA) microRNA (miRNA) expression datasets used in the validation experiment.

Tumor Type	Abbreviation	Number of Samples
Sarcoma	SARC	263
Esophageal carcinoma	ESCA	198
Acute myeloid leukemia	LAML	188
Pheochromocytoma and Paraganglioma	PCPG	187
Pancreatic adenocarcinoma	PAAD	183
Rectum adenocarcinoma	READ	165
Testicular germ cell tumors	TGCT	156
Thymoma	THYM	126
Kidney chromophobe	KICH	91
Mesothelioma	MESO	87
Adrenocortical carcinoma	ACC	80
Uveal melanoma	UVM	80
Uterine carcinosarcoma	UCS	57
Lymphoid neoplasm diffuse large B-cell lymphoma	DLBC	47
Cholangiocarcinoma	CHOL	45
Glioblastoma multiforme	GBM	5

**Table 3:** Top 10 miRNAs by feature importance and Pearson correlation coefficient (PCC).

Top	miRNA	Feature-Importance Score	PCC
1	hsa-mir-155	0.2309	−0.6037
2	hsa-mir-4772	0.0899	−0.5834
3	hsa-mir-142	0.0575	−0.5111
4	hsa-mir-150	0.0330	−0.5744
5	hsa-mir-223	0.0225	−0.5003
6	hsa-mir-200c	0.0211	0.0398
7	hsa-mir-141	0.0159	−0.0197
8	hsa-mir-200b	0.0153	0.0048
9	hsa-mir-92a-1	0.0134	0.1800
10	hsa-mir-22	0.0120	−0.3558

**Table 4:** Kyoto Encyclopedia of Genes and Genomes (KEGG) pathways enriched by the target genes of miRNA top-ranked by the random forest regression’s feature-importance score (adjusted *p*-value < 0.01).

KEGG Pathways	Adjusted *p*-Value
Interleukin (IL)-17 signaling pathway	0.0014
Pathways in cancer	0.0014
Hepatitis B	0.0014
T-cell receptor signaling pathway	0.0015
Tumor necrosis factor (TNF) signaling pathway	0.0021
Osteoclast differentiation	0.0043
Mitogen-activated protein kinase (MAPK) signaling pathway	0.0052
Toll-like receptor signaling pathway	0.0052
Lipid and atherosclerosis	0.0052
Pancreatic cancer	0.0053
Signaling pathways regulating pluripotency of stem cells	0.0053
B-cell receptor signaling pathway	0.0064
Colorectal cancer	0.0082

## Data Availability

Publicly available datasets were analyzed in this study. This data can be found here: https://xenabrowser.net/.

## Supplementary Materials

The following supporting information can be downloaded at: https://www.mdpi.com/article/10.3390/biology11050787/s1, Table S1: Optimal parameters using a grid search with 10-fold cross validation (GridSearchCV); Table S2: Target genes of hsa-miR-155; Table S3: Target genes of top three miRNAs (hsa-mir-155, hsa-mir-4772, hsa-mir-142); Table S4: KEGG pathways enriched by the target genes of miRNA top-ranked by the random forest regression’s feature-importance score (adjusted p-value < 0.05); Table S5: KEGG pathways enriched by the target genes of the top three miRNAs (adjusted *p*-value < 0.05); Table S6: GO Biological Process enriched by the target genes of miRNA top-ranked by the random forest regression’s feature-importance score (adjusted *p*-value < 0.05); Table S7: GO Biological Process enriched by the target genes of the top three miRNAs (adjusted *p*-value < 0.05).
